# Theoretical research on specific human resources in tourism. Features in Romania

**Published:** 2014

**Authors:** Carmen Cristina Albu, Dan Constantin Vărzaru

**Affiliations:** * Transilvania University Braşov, Brașov, Romania; ** University of Craiova, Craiova, Romania

**Abstract**

Tourism is a sector of the economy in which the problems related to the jobs and necessary skills to exit the crisis are emphasized more sensitively than in the other sectors of the economy. This paper proposes a literature review based on the latest studies and research literature worldwide (USA, Canada, EU, etc.), aims to identify the factors and trends influencing skills shortages in tourism and also the policies to mitigate this deficit. Such an approach is not easy, as most specialists in tourism admit a chronic insufficiency of the research and of the information specific to this domain of activity.

Tourism feels the chronic global shortage of human resources due to its seasonal activity and its lower efficiency. The solutions that are mostly being relied upon take into consideration the closer involvement of the Government in tourism support.

An important role lies in defining the content engine training and education. The differences between the requirements of the tourism sector and the content of vocational, technical and university learning is a problem in many countries. The situation remedy requires program rethinking, teachers improving skills, creating practical programmes in terms of quality, preference in enterprises and establishment of the most appropriate bridge linking vocational (professional) education and higher education to open the students' clear and open opportunities. Even in countries where tourism responsibilities are divided between different ministries and agencies, a strategy of national long-term growth in this area is essential.

The paper lists the types of partnerships between public powers, the tourism sector and the educational sector at a global level and highlights their potential in connection with national and cultural specifics.

**Keywords:** human resources, tourism, economy

